# Development and Experimental Comparison of Low-Cost, Reliable Capacitive Touch Sensing Boards

**DOI:** 10.3390/s16111786

**Published:** 2016-10-26

**Authors:** Ferat Akkoç, Özge Cihanbeğendi Şahin

**Affiliations:** 1 Siemens AS, Corporate Technology Development Center, Kocaeli 41480, Turkey; 2 Department of Electrical and Electronic Engineering, Engineering Faculty, Dokuz Eylul University, Izmir 35390, Turkey; ozge.sahin@deu.edu.tr

**Keywords:** capacitive touch sensors, mutual and self-capacitance, charge transfer, noise detection, frequency hopping

## Abstract

In this study, two types of direct interface capacitive sensors, self- and mutual-capacitance, were developed and compared experimentally. Electromagnetic Compatibility (EMC) tests—International Electrotechnical Commission (IEC) 61000-4-3, IEC 61000-4-4, IEC 61000-4-6—were applied in an accredited laboratory to measure the immunity of the sensors against radiated and conducted interference. The frequency hopping algorithm could be implemented for the mutual-capacitance sensor without using any particular circuit. The effects of EMC disturbance were detected by means of a new noise detection algorithm and when the signal-to-noise ratio (SNR) became lower, the operation frequency of the sensors switched to an undisturbed frequency to ensure safe operation. For this purpose, a new noise detection algorithm was developed and frequency hopping was performed with a standard controller. Both cards were tested under several conditions and their performances compared.

## 1. Introduction

Capacitive sensors currently find a wide range of applications in many areas including consumer electronics, industry, and automotive sectors. In this respect, the most important factor is the unique relative permittivity (dielectric constant) ($\varepsilon_{r}$) which directly affects capacitance and is influenced by environmental conditioning. There are considerable numbers of ways of measuring the capacitance change [1]. One of the sensors designed for measuring capacitance change is the touch sensor.

The capacitance variations measured at sensors are quite small and the values obtained are extremely weak, in picofarad (pF) range, or sometimes even below. They are rather sensitive to interference and easily affected and disrupted by electric noise originating from nearby devices, neighboring sensor wires (coupling) or from the power grids(conducted noise). The power grids are the most troublesome interference source [2]. Furthermore, since both ambient temperature and humidity change $\varepsilon_{r}$ this affects the signal levels [3,4]. To cope with these sorts of drawbacks, many sensor researches have been conducted. Especially, some modules and circuit designs that combine with analog and digital components are used. For instance, the chip AD7745 capacitance-to-digital converter of the Analog Device includes a sigma-delta modulator (Σ-Δ), a third-order digital filter, and excitation source modules [5]. A chip of the PSOC family of Cypress Semiconductors includes a CapSense (CSD) module with pseudo random generator (PRS) and analog-digital modules [6]. The MSP430 family from Texas Instruments features CapTIvate technology which contains analog-digital components to achieve noise immunity [7]. Microchip Technology offers the slew-rate limiter technique against impulse pulses [8]. Up to a point, it contributed to our performance; however it could not give sufficient contribution on its own to succeed for conducted noise. Yu and Sun [9] offer an inverse charge-transfer method including a reference channel for Atmel QTouch (self-capacitance) in case of noisy power. Since QTouch succeeded in electromagnetic compatibility (EMC) tests without trouble in our study, we compared it experimentally with Atmel QMatrix (mutual-capacitance). Another successful method is the correlated double sampling (CDS) algorithm to cancel common-mode noise by saving a noise reference pattern for mutual-capacitance [10]. In addition, a numerically controlled oscillator (NCO), operational or charge amplifiers, and analog switches are usually used to reduce internal and external noise [11,12,13]. Unfortunately, the extra modules or component used raise the price of the microcontroller unit (MCU). Moreover, they are only used for touch sensing, namely in a project a second MCU must be used to handle general tasks. This also increases the BoM (bill of materials) of the control card. In this study, it cost in the order of 5–7 $ for each touch sensing board.

On the other hand, considering no analog and digital components are used, the signals are rather noisy; consequently sensors suffer from parasitic capacitances, interference, and RF emissions which results in inconsistent results. Parasitic capacitances originate from the layout of the card and the wiring lines [14]. Delta-sigma is a highly popular technique to deal with parasitic capacitance [15]. Some techniques such as active shielding reduce the coupling interference via air [16]. When noise comes from the power grids or is injected into power lines (conducted noise), it affects the pins of the MCU and sensor wires, so it creates slight voltage fluctuations. Such an extraordinary situation can be simulated through EMC tests; Active shielding or hardware filters prevent only the coupling effect originating from nearby sensors or wires, and not conducted noise. To overcome the noise, a customized IC (AD7745, MSP430) or high-end MCU (Cypress) containing special module is used, but it increases the cost.

This paper presents a novel method of decreasing the conducted noise. It can be used in any low-end MCUs which do not need any particular analog- and digital modules. Firstly, a novel noise detection algorithm was developed to detect EMC disturbance. Comparing the noise detection algorithm with the standard deviation, shows that it consumes very small MCU resources. The standard deviation formula is a common method to identify disturbances and consumes a great deal of time and resource. When the noise is detected, the frequency hopping is realized. So, the operating frequency of the sensors is swapped to an undisrupted band. Frequency hopping is implemented without using a special module (PRS) and clock source (excitation); only a resistance-capacitance (RC) oscillator is used. Both a simple analog-digital converter (ADC) and an RC are common modules for each controller. In order to investigate the effects of this method on capacitive sensors, two different touch sensing cards with the charge transfer method were designed only for testing, and the software was developed on the basis of the cards. This paper also presents experimental analysis and performance results.

## 2. Theory of Capacitive Touch Sensing Methods

### 2.1. Charge Transfer Method

The charge transfer method was implemented with a switched-capacitor (SC) technique in order to measure relative capacitance variation at the sensors when touched.

**Figure 1:**
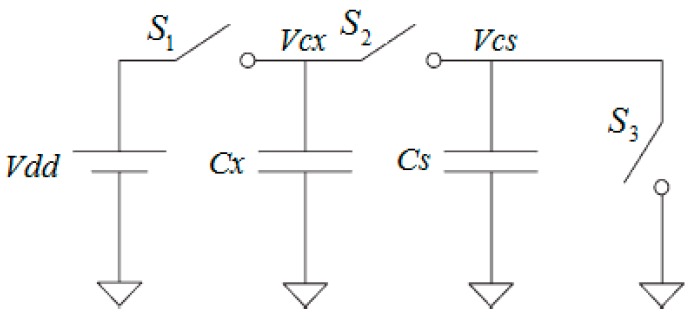
A simple circuit of the switch-capacitor technique.

Figure 1 shows the operation of a basic SC branch which was reported by Gaitán-Pitre, Gasulla, and Pallàs-Areny [14]. A series of voltage pulses V_dd_ is applied to an unknown capacitance *C_x_* and a sampling capacitor *C_s_*. By opening and closing *S*_1_, *S*_2_, and *S*_3_ switches respectively, two non-overlapping phases take place. In the first phase charge is accumulated on the *C_x_* and in the second phase the charge stored on *C_x_* is discharged into the larger *C_s_*. After repeated transfer cycles, the voltage across *C_s_* is compared to a fixed reference voltage. This circuit demonstrates how the charge transfer method works theoretically.

Taking Figure 1 into account the voltage across *C_s_* is

(1) $$V_{cs}\left[n\right]=\frac{C_{x}}{C_{x}+C_{s}}Vdd+\frac{C_{s}}{C_{x}+C_{s}}V_{cs}\left[n-1\right]$$

where ‘n’ is charge-transfer cycles, *V_cs_* is voltage after n cycles, and *V_dd_* is the pin voltage of the MCU.

When *C_x_* and *C_s_* are in series the charging current flowing in the capacitors will be equal. This current charges the capacitors equally without taking the capacitor values into account.

(2) $$C=\frac{Q}{V}\text{ and }V_{dd}=V_{cx}+V_{cs}$$

(3) $$V_{dd}=\frac{Q}{C_{x}}+\frac{Q}{C_{s}}\text{ (Kirchhoff’s Voltage Low)}$$

(4) $$V_{cs}=\frac{C_{x}}{C_{x}+C_{s}}Vdd\;(C_{x}\text{ and }C_{s}\text{ voltage dividers})$$

(5) $$\frac{C_{x}}{C_{s}}=\frac{1}{1000}\;(\text{approximately},C_{s}>>C_{x})$$

*C_x_* is now charged to 99.9% of *V_dd_* in the first charge-transfer cycle.

### 2.2. Mutual-Capacitance Sensor Theory

Mutual-capacitance results from the potential difference between two conductive plates. The electrodes are positioned; PCB and air are the kinds of dielectric substances between them.

**Figure 2:**
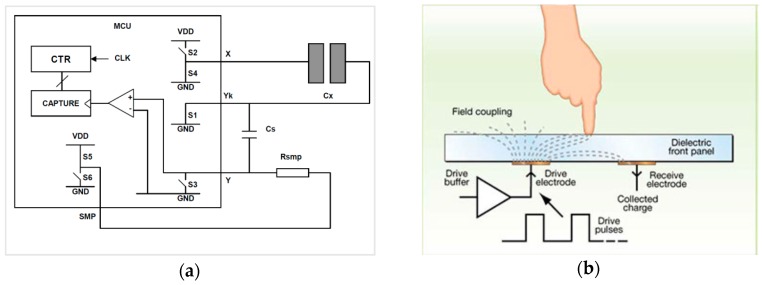
(**a**) The equivalent circuit in measurement phase [17]; (**b**) field coupling between electrodes. Dielectric front panel is FR4 PCB in the study. In products, usually, a glass is also put on the printed circuit board (PCB).

Figure 2a shows the equivalent circuit which uses only a few components and which is easily implemented with any MCU. Each sensing electrode pair contains a drive and a receive electrode. The drive electrode (X) is driven by logic pulses; the receive electrode (Y_k_) collects most of the charge that is coupled via the PCB and air. Since the human body conducts away a portion of the field, the field coupling is attenuated by a finger touch (Figure 2b). Typical pulse time is from 250 ns to 2 µs and the number of pulses is from 16 to 64.

**Figure 3:**
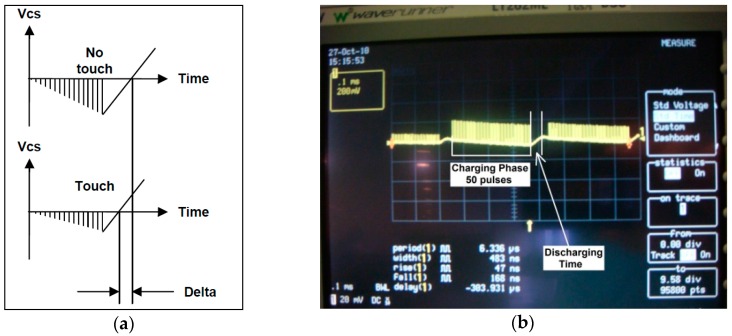
(**a**) Theoretical demonstration when a finger steals some of amount charge from the sensor; (**b**) Charge-discharge phase with 50 pulses.

When a finger gets closer to the sensor, the finger capacitance becomes in parallel with the sensor capacitance *C_x_*. Therefore, each pulse charges *C_s_* by a smaller amount. Then *V_Cs_* rises more slowly and the maximum charge level at *C_s_* is less than the untouched state. So, less time is required to discharge *V_Cs_* because the discharging part of the circuit is never changed (Figure 2a). Figure 3a compares the touched and untouched state. ‘Delta’ is shown as the change in counting the discharge time. During normal operation, if the Delta value is at least 10–15 counts, it is accepted that a touch is detected. The method is highly stable and insensitive to changes in *V_DD_* and *C_s_* [17].

Table 1 lists the sequence diagram of charge-transfer cycles including the switch states (Figure 2a). *C_x_* is discharged at State #7 since *S*_1_ and *S*_4_ switches are closed. Then the loop restarts after having returned to State #2.

### 2.3. Self-Capacitance Sensor Theory

**Figure 4:**
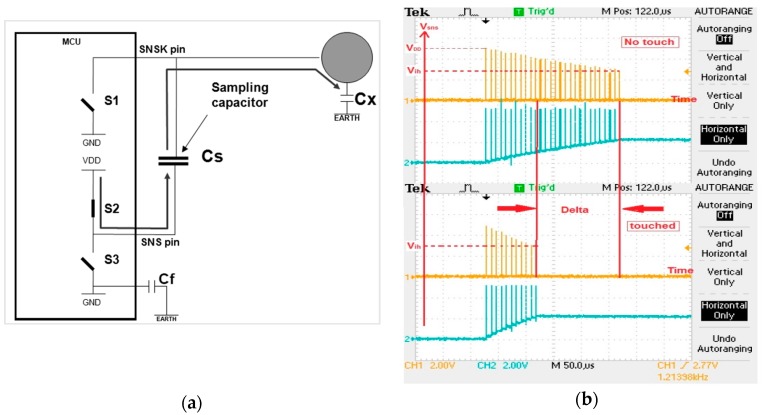
(**a**)The equivalent circuit in charging phase; (**b**) Voltage waveforms across *C_s_*.

This method uses a single electrode by taking earth ground as a basic element. This electrode can be considered both as transmitter and receiver. Figure 4a shows the equivalent circuit. The circuit and its operation are not as complex as the mutual-capacitance sensor. It uses only two digital Input-Output (I/O) pins and a sampling capacitor—*C_s_*. In the design *C_s_* >> *C_x_*; such that *C_s_* is of the order of nanofarads (1–100 nF in most applications) where *C_x_* is of the order of picofarads. *C_s_* is selected as 4.7 nF.

In this method, both the charge and the measurement phase take place on each charge-transfer loop. This loop continues until *C_s_* reaches 5V and the MCU sense logic ‘1’on the SNSK pin. When a finger gets closer to the sensor, each pulse charges *C_s_* by a large amount and *C_s_* reaches 5 V quicker by a few pulses. After that we count how many loops it takes to charge *C_s_* up to 5 V, which is *V_ih_*, the digital input threshold. The end result is to compare the number of pulses with the untouched case and decide whether a finger has touched. It can be clearly seen with Figure 4b that the *V_ih_* is obtained with less pulse in case of touch. ‘Delta’ refers to how few are the numbers of pulses transferred when one touches the sensor.

The operation sequences are shown in Table 2. State #3 is the charge phase and state #5 refers to the measure phase. This loop continues until the SNSK pin reaches *V_ih_*. The number of loops is directly proportional to *C_s_/C_x_*. This technique allows high-resolution measurement of pF-level capacitances.

## 3. Design of the Sensor Cards

### 3.1. Hardware Design

To make the tests experimentally comparable, two identical cards were designed only with different sensor shapes. The used components, the layouts of PCB, the processors (ATmega329P), software architectures, and sensor libraries were all the same. Both cards consist of 10 single buttons and one slider sensor which is an array of single sensors. The MCU operates at 8 MHz for self-capacitance and the clock source is an external crystal oscillator (XTAL). The MCU operates at various frequencies for mutual-capacitance and the clock source is an internal oscillator (RC) to realize frequency hopping.

Some guidelines were followed while designing the sensing cards and can be summarized as follows:

- *C_x_* and *C_s_* capacitors are connected to each other through serial resistance. This refers to the copper trace resistance between these two capacitors. The effect on the RC time constant needs to be monitored. Normally, serial resistance improves Electromagnetic Interference (EMI) and Electrostatic Discharge (ESD). Therefore, 1 kΩ resistors are added to some designs [17].
- *C_s_* is the most important component of the measuring circuit. The circuit includes a ceramic capacitor of type X7R which has low tolerance value against temperature.
- The 7805 regulator IC is used for obtaining a linear regulated supply.

All sensors in the mutual-capacitance card are constructed on the bottom layer of the PCB. The top layer does not include any sensors or their traces. Therefore, we touch on the top layer. PCB can be thought as a substrate or cover. Normally, a glass is assembled on the top. There are 16 channels which are connected in a matrix of 8_×_2. The buttons consist of one channel whereas the slider includes six.

**Figure 5:**
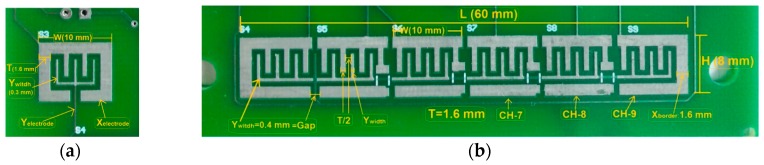
The shape of an electrode of mutual-capacitive (**a**) single sensor; (**b**) slider on the bottom layer.

The single sensor is a simple on/off touch button. The X and Y electrodes for each channel are interdigitated comb electrodes, which are a very common type in sensing applications. Typically the X electrode surrounds the Y electrode, as it helps to contain the field between the two electrodes. The other advantages of interdigitated design are to optimize the SNR value by maximizing the coupling length between the X and Y electrodes, to increase the sensing area and so on [18] (see Figure 5a). The slider on the card having six channels is 6 cm long, in which the channels are laid together side by side. There are no borders between the channels while borders exist on the edges of the slider. Figure 5b illustrates the structure of a slider.

**Figure 6:**
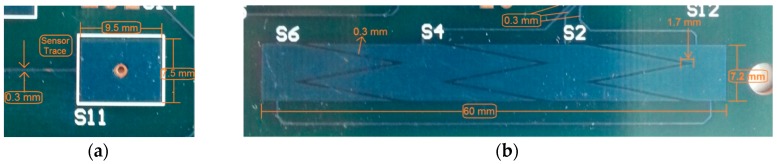
Construction of electrodes of self-capacitive (**a**) single sensor; (**b**) slider.

A self-capacitance sensor consists of a single-plate electrode which is formed on a single layer. As it is simpler, there is not much limitation with regard to the dimension of the electrodes which have a square shape (Figure 6a). In the slider design, the electrodes on the left and right side are half and they two represent a single electrode as a whole. These are regarded as toothed electrodes which are used instead of square electrodes in order to increase resolution when the finger moves (Figure 6b).

### 3.2. Software Design

During the design process of sensors some basic limitations should be taken into account. The first and the most important one is SNR. If it is low in spite of all developments and enhancements, software filters are used in order to prevent false detection. Other limitations are the memory of the processor and the response time. In such applications, the software filters should have small code sizes and include fast filtering techniques.

There are many studies in the literature to increase SNR and to filter noise. For instance, according to the study of Chou, et al. [19], the signals received are transported to a high frequency band by using a modulator. Then, the signals are increased by means of an amplifier and the increased signals are demodulated and passed through a low pass filter (LPF). Noise signals are reduced at the output of the LPF, so the SNR is increased.

Some software filtering techniques are applied consecutively to pass some EMC tests and to have a more reliable and fast measurement process. The flowchart diagram and the filter structures are given as (Appendix B).

The first filter oversamples to prevent false detection. This may be thought as a debouncing time as for hard-buttons. This provides more acquisition samples per reading of sensors. There may bea false key detection on the card due to electrical noise. Especially, high impulses are injected onto the card during the Electrical Fast Transient (EFT) tests. In this case, it may be difficult to decide whether the sensor is detected or not only by a single acquisition sample. Therefore, there should be more than one acquisition in all sensors.

The second one is the Slew-Rate Limiter (SRL) to reject impulse noise and smooth the signal. When a new reading value is generated, it is compared to the current reference value. If it is much higher or lower than the reference, it is accepted accruing as a result of the noise and so discarded. Instead, the reference is then either decremented or incremented by ‘1’. So, very large and very small values occurring as a result of the noise are discarded.

The last one is a running average filter which is defined as a type of finite impulse response (FIR). This is defined from the formulae in Equations (6) and (7);

(6) $$y\left[n\right]=\frac{1}{L}\left\{x\left[n\right]+x\left[n-1\right]+\ldots +x\left[n-L+1\right]\right\}$$

(7) $$=\frac{1}{L}\sum_{l=0}^{L-1}x\left[n-L\right]$$

where the output signal y[n] is the average of L input samples.

According to Equation (3), L memory buffers are required to place x[n],x[n−1],…,x[n−L+1] samples and also L-1 additions are needed. The filters are used to smooth signals when the EMC test fluctuates the original signals [20].

## 4. Noise Reduction and Frequency Hopping

Capacitive sensors are highly sensitive to both electrical noise and environmental changes when devices are particularly supplied by power grids. Although there are several reasons why noises are seen on sensors the most important one is that they are analog. Measured signals can easily be affected by ambient conditions. If they were digital sensors, the state of the MCU pins would only be in high or low states. Another reason is when a user touches the sensors; the situation user becomes a part of the sensor circuit. The user acts as ground which is different from the reference of the sensors. At this time, the sensors have two different ground references and they may interpret it as an injected noise [8].

To reduce the effects of environmental noise and EMC disturbance, several basic noise suppressions have already been investigated. Some of them are ratiometric measurement [2,21], the modulation/demodulation system [19], combined frequency selection [22], the Decentralized Kalman Filtering (DKF) approach [23], and the frequency hopping approach [6,24]. These kinds of solutions usually require a specific circuit design which includes both analog and digital components. It increases not only the cost, but also the complexity of the design.

References [8,14] propose a low-cost direct interface circuit based on charge transfer. Unfortunately, they do not mention any consequences of EMC tests except the work in [8] which offers a slew-rate limiter against impulse noise and is mentioned in the introduction. This paper presents more robust and reliable direct interface sensors by using the frequency hopping technique and by detecting the disturbance without using an extra readout circuit.

Frequency hopping has widely been used to increase SNR and for operating the sensors on noiseless frequency bands in capacitive systems. When SNR becomes lower, sensors switch to an undisturbed frequency band. However, this method raises two important problems when standard, resource-limited MCUs are used.

The first one is how the carrier frequency of the sensors can be changed. Sensors are directly connected to the MCU and are driven as logic high and low pulses. The matter is dealt with by an internal RC oscillator. It provides an 8 MHz clock approximately. Although it is voltage and temperature dependent, it can accurately be calibrated by using the oscillator calibration register (OSCCAL). By changing the OSCCAL in the software, it is possible to change the frequency of the processor. Thus, there is no need for external circuitry as stated. Three frequencies are used in this study. The base frequency is f_c1_ when there is no disturbance. Table 3 shows the frequencies with their OSCCAL values and the CPU (RCclk) clock. Normally, frequency hopping is realized in a very narrow frequency band range which is a bandwidth of 20 kHz. This helps to prevent disturbance based on EMC.

The microcontroller has many peripheral modules which are UART, SPI, I^2^C, and timers. Their individual operating clocks depend on the MCU clock. So, module registers have to be set simultaneously when the MCU clock changes. Therefore, we calculated all register values where modules work at each frequency properly. Otherwise, communication will be interrupted or there will be a shift of time in the timers. In this way, we no longer need a specific circuit to do frequency hopping.

The other problem is detecting the noise. If it originates from the power grids, it is expected to affect all channels. Therefore, all channels have to be examined to monitor it with its strength. The most common detection algorithm is to calculate standard deviation. Brasseur [2] and Kerö, Nachtnebel, Pommer, and Saute [22] detected EMC problems by comparing the standard deviation of 50 consecutive measurement values with the average of the same data. When noise occurs, the noise strength reaches a specific ratio (r=σ/μ) and then the carrier frequency is altered to an undisturbed band. In our design, each measurement value is 2 bytes. If 50 values are obtained and recorded for every channel, the number of bytes required in RAM would be:

16(channels) × 2(bytes) × 50 = 1600 bytes.

The RAM for ATmage329P is 2048 bytes. If the standard deviation is performed on this basis, it consumes a large amount of memory, execution time, and power. Sauter and Nachtnebel [24] modified the standard deviation and developed an easy-to-implement algorithm for a simple controller. Watzenig, Steiner, and Zangl [23] proposed the decentralized Kalman filtering technique by reducing computational complexity for one iteration of the Kalman algorithm. If these modified algorithms were applied to all channels, the resources would be still insufficient. Therefore, we developed a new detection of the interfering algorithm which has a small code size and is very efficient when so many channels are used.

The algorithm includes two kinds of calculation. The first one (Equation (8)) obtains the current measurement values of all channels and compares them with the previous ones to detect noise strength. It no longer needs to store 50 consecutive values for each channel, but instead it obtains the values from 16 channels and detects their correlation towards noise. One advantage of the filter is that each channel records only the previous value in the memory. Therefore, it does not consume so many bytes and the calculation speed is quite high.

(8) $$x\left[n\right]=\frac{1}{CH}\overset{CH-1}{\underset{k=0}{\sum }}\left|\Delta k\left[n-1\right]-\Delta k\left[n\right]\right|$$

where:

x[n] is the average channel noise strength
Δk[n] is the channel delta at time ‘n’
Δk[n−1] is the channel’s previous delta
|Δk[n−1]−Δk[n]| is the delta at k channel
k is a channel counter variable and CH is the number of channel.

The channel noise strength, x[n] evaluated for all channels is an input argument of the infinite impulse response (IIR) filter along with the previous noise ratio—y[n−1] (Equation (9)). The result obtained from the IIR filter is called the noise ratio, y[n], and is defined as:

(9) $$y\left[n\right]=y\left[n-1\right]+\frac{x\left[n\right]-y\left[n-1\right]}{L}$$

where:

y[n] is the noise ratio (the intensity of disruption)
y[n−1] is the previous noise ratio
L is noise strength size

**Figure 7:**
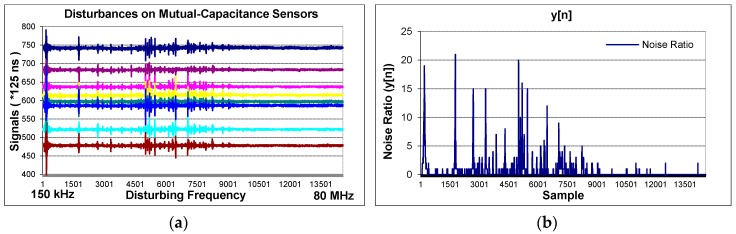
(**a**) Signals are received from 8 channels when the noise being injected differs between 150 kHz and 80 MHz; (**b**) the output of the noise detection algorithm.

The noise ratio y[n] is a quantity that shows the intensity of disruption on the sensors. The effect of EMC is illustrated in Figure 7a. The carrier frequency of sensors is at 158 kHz and the frequency hopping is forbidden. Noise injected differs between 150 kHz and 80 MHz. When the carrier frequency or its harmonics overlap with the noisy frequencies, the sensors are disturbed. This effect is decreasingly ongoing with high harmonics. At the same times, the disruption intensity can be seen in Figure 7b. It shows the output of the noise detection algorithms which works in the background simultaneously. In this test, frequency hopping is forbidden but in the next part, the test is repeated by applying frequency hopping. We limit this quantity as a “3”. Whenever the noise ratio reaches “3”, frequency hopping is realized.

## 5. Measurement Results and Discussion

### 5.1. Frequency Hopping Tests

**Figure 8:**
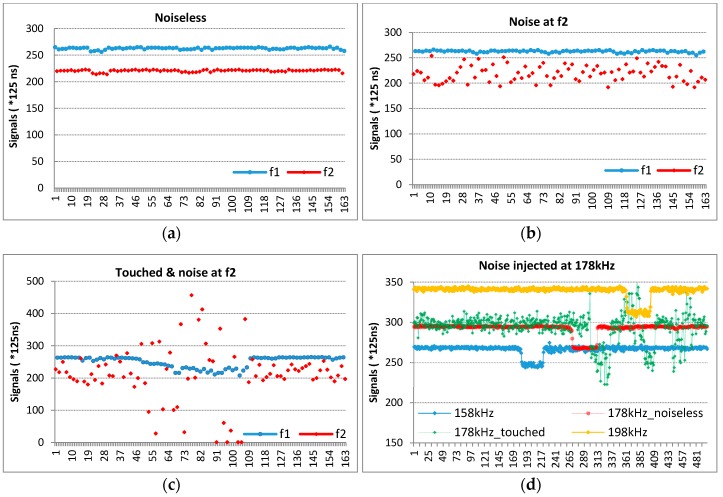
The effect of injected noise on the mutual-capacitance sensor; (**a**) the noiseless frequencies; (**b**) the injected noise at f_2_ frequency; (**c**) touching on a sensor while injecting noise at f_2_ frequency; (**d**) while injecting noise at 178kHz, channel-6 is driven by the carrier at 158 kHz, 178 kHz and 198 kHz respectively.

A sensor is driven at two frequencies f_1_ and f_2_ in turn such as f_1_-f_2_-f_1_-f_2_-f_1_-f_2_ (Figure 8a). Then signals are saved by injecting noise at f_2_. Although the sensor is driven at f_1_ and f_2_ simultaneously, signals at f_2_ are disrupted; by contrast, signals at f_1_ are not (Figure 8b). Normally, the noise varies between 150 kHz to 80 MHz. We injected noise at a constant frequency (f_2_) to show its effect on the sensor when driven at f_2_.

Later, while continuing to inject noise, the sensor was touched (Figure 8c). With the effect of the finger, while a proper decrease occurred at a noiseless frequency (f_1_), disruption occurred at a noisy frequency. This is because, as mentioned above, that system has two different ground references.

Figure 8d shows channel-6 in different cases. Channel-6 is driven at 158 kHz, 178 kHz, and 198 kHz respectively while injecting noise just at 178 kHz and touching simultaneously. When carrier frequencies are at 158 kHz and 198 kHz the button is not affected by touching, the carrier The 178 kHz is affected as indicated in the green line (178 kHz_touched). Normally, it must be as the red line.

**Figure 9:**
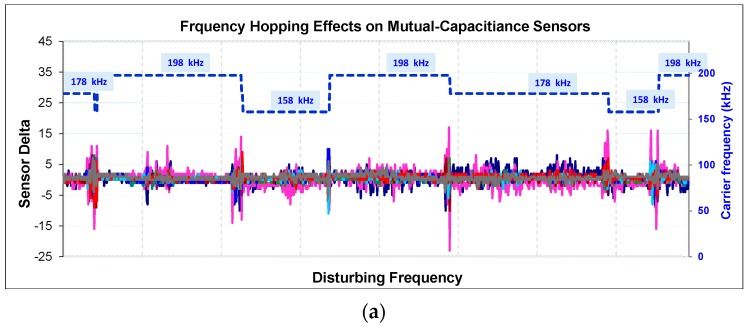

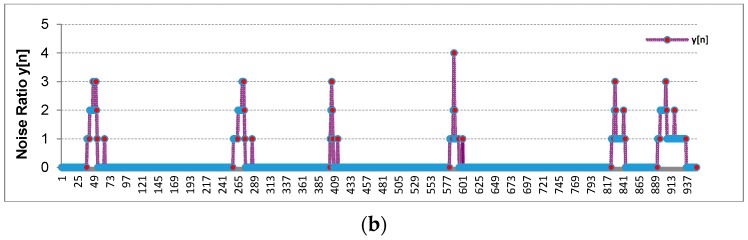
illustrates the frequency hopping effect for all sensors. When y[n] reaches **3**, the carrier frequency is altered to a noiseless band (blue dash-line). (**a**) The disturbed mutual-capacitance sensor; (**b**) the noise ratio.

The noise detection algorithm should run for checking not only one-channel but also all channels fast and effectively without raising any response time. Additionally, it should trigger frequency hopping to jumps to an undisturbed carrier. Figure 9 shows this situation for all ‘Delta’ values of sensors, not channels. As is seen in Figure 9a, all signals are becoming worse and are distributed simultaneously; the algorithms detects the disturbance; when the noise ratio reaches to 3 or over 3 (Figure 9b), the carrier is changed and signals are carried to an undistributed band.

### 5.2. Temperature and Humidity-Droplet Tests

In this part, the immunity of sensors against environmental condition changes such that temperature and humidity-droplets are being tested and compared and their performances evaluated.

First, the effect of ambient temperature was examined. Temperature affects the ε_r_ coefficient. Depending on ε_r_, the sensitivity factor S = ε_r_/t (t is thickness) changes for one layer. An increase in temperature increases the dielectric constant of fiberglass reinforced epoxy (FR4 PCB) and the electric field starts to propagate easier and better in PCB. We used FR4 PCB. Hinaga, et al. [25] investigated the increase in dielectric constant of FR4 types with temperature and concluded that the percent change of various type of FR4 laminates between 23 °C and 75 °C ranges from 0.26% to 8.98%. Therefore, an increase in sensitivity factor of the sensors is expected. Now each pulse makes the sampling capacitor charge more.

**Figure 10:**
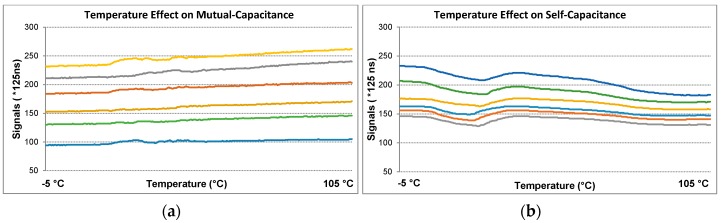
The effect of temperature change (**a**) for mutual-capacitance; (**b**) for self-capacitance.

Mutual sensors use a dual-slope conversion method that means charging and discharging a sampling capacitor *C_s_* (Figure 3) in the opposite directions respectively since the voltage across *C_s_* mainly depends on the driven voltage (*V_DD_*) of the pin when untouched. Thus, it is more stable to the changes in temperature. Also this change is less since the measurement of mutual coupling is realized in a small local area and both electrodes are affected at the same rate. The tests are done from −5 °C to +105 °C. Figure 10 shows test results for the both cards. The maximum signal change rate is 13.75% for mutual sensors whereas it is 21.46% for self-sensors. It can be concluded that mutual sensors are more stable to temperature than self-sensors. Ambient temperature does not lead to false detection, because drift and filter algorithms can track signals easily and update the current reference values.

One of the serious problems in capacitive sensors is humidity and water films. As the humidity increases, the dielectric constant increases and the signals increase. If this change is just caused by ambient humidity, it can be compensated with drift algorithms. If it is so fast, drift algorithms cannot detect it and false detection occurs. The only way to overcome this problem is to fasten the drift mechanism. Although very fast drift overcomes the problem, when a finger closes to a sensor, the sensor drifts very fast according to the finger and this time it will not sense the finger. Since humidity usually changes slowly, it can be tracked and compensated, so sensors can change their reference slowly according to current values. Therefore, in this part the water film effect is examined.

The most important effect of water films—droplets—is false detection. Because water contains dissolved ionic molecules and they allow electrical conduction. It has an effect as if a finger is touching. This results in false detection.

**Figure 11:**
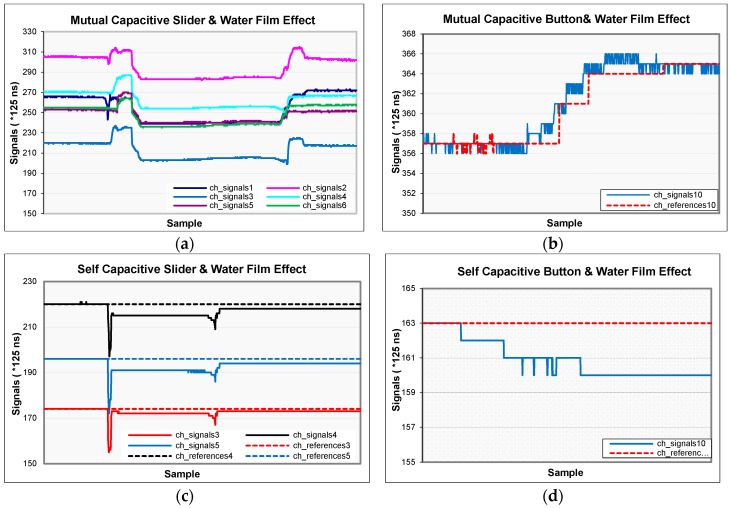
Signal changes as a result of water film test on two types of sensors: (**a**) The slider is composed of 6 channels; (**b**) on the 10th channel; (**c**) the slider consists of 3 channels; (**d**) on the 10th channel of self-capacitance sensors. (b) and (d) are related to a single button.

In Figure 11, water film test results are shown for the sliders and single buttons of both cards. All signals decrease at every slider channel for both types of sensors (Figure 11a,c). Because slider channels are side by side and when a pulse is sent to a channel, other channels are grounded. The electric field that propagates from one channel goes through the other channels grounded over water. So less charge reaches the receiving electrode (see Appendix A).

Each water drop on the buttons increases the mutual coupling between X and Y electrodes and the coupled electric field increases [18]. So the acquired signal increases (Figure 11b). This does not cause false detection because the human finger decreases the signal levels by absorbing some of the charge. It causes an adverse effect according to a finger. This attribute shows that the mutual-capacitance sensors have a natural moisture suppression feature. However, each water drop in the self-capacitive sensors increases the capacitance of the electrode to the earth. Thus, it causes the sampling capacitor to charge with fewer numbers of pulses (Figure 11d). In spite of the signal change rate being less in self-capacitance sensors, this may rarely result in a false detection. The drift mechanism is turned off during the tests to show the effect of droplets.

### 5.3. Electromagnetic Immunity (EMC) Tests

The immunity tests are divided into two parts; conducted and radiated. Conducted noise occurs on devices which are supplied by power grids. Radiated noise originates from some devices working at high frequencies such as mobile phones or high-power communication lines.

**Figure 12:**
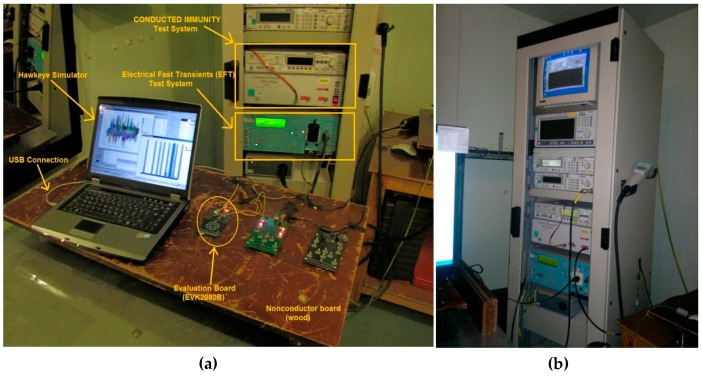
Measurement set-up with EFT and conducted immunity test devices.

Some EMC tests are related to power supply cards. They test the immunity of cards to voltage fluctuation in power grids. Instead of designing a new power supply, an EMC approved CE mark carrying 9 V adapter was used during the tests. Therefore, only some of the EMC tests were applied. The test laboratory used is accredited by international test organizations. Taking the certificated laboratory regulations into account, related tests; in information technology equipment IEC 61000-4-6, IEC 61000-4-4, IEC 61000-4-3 [26,27,28] were applied. Figure 12a shows the measurement set-up including EMC instrumentations. The EVK2080B gathers sensor data and sends it to the Hawkeye simulator via a USB cable in the proper order. It also acts as an optocoupler to protect USB inputs against high voltage.

**Figure 13:**
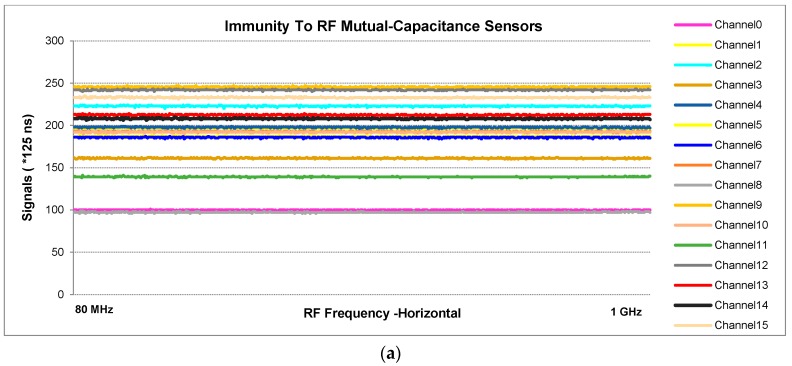

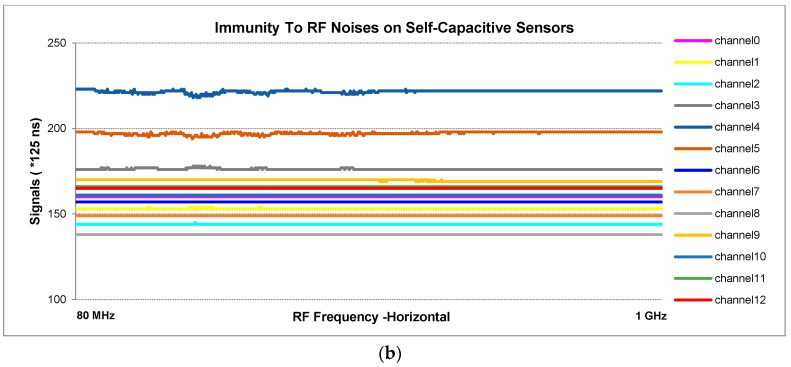
Radiated immunity test to radiated, radio-frequency and electromagnetic fields for: (**a**) mutual-capacitance; (**b**) self-capacitance.

The first test is the electromagnetic radiation (IEC 61000-4-3). This radiation is frequently generated by such sources as radio or television transmitters, mobile antennas and so on (Figure 13).

The next test relates to repetitive electrical fast transients (EFT). It is a burst consisting of a number of fast transients. Significant issues for the test are high amplitude, short rise time, high repetition rate, and low energy of the transients (IEC 61000-4-4). Because these high voltage impulses affect all the measurement set-ups, we could not get comprehensible results. We observed that the noise was filtered by soft-filters and any undesirable touch did not occur.

The last test relates to the electromagnetic disturbances coming from RF transmitters, especially field strength of up to 15 V/m in a frequency range 150 kHz to 80 MHz in information technology equipment (IEC 61000-4-6). The main goal of this test is to measure immunity for electronic components inside the product against the electromagnetic interference conducted from the cables and the effects of RF interference on the cables [28] (The test detail is given in Appendix C).

This test is more important than previous ones. Because other tests can be filtered by hardware and software filters. This injected noise can manage to pass through both power supply and the filters, so directly affects the sensors. It cannot be filtered by standard filters. Nevertheless, the injected noise affects all channels simultaneously, so it gives us an opportunity to determine it when it comes. Thanks to this effect, the noise detection algorithm could be developed.

**Figure 14:**
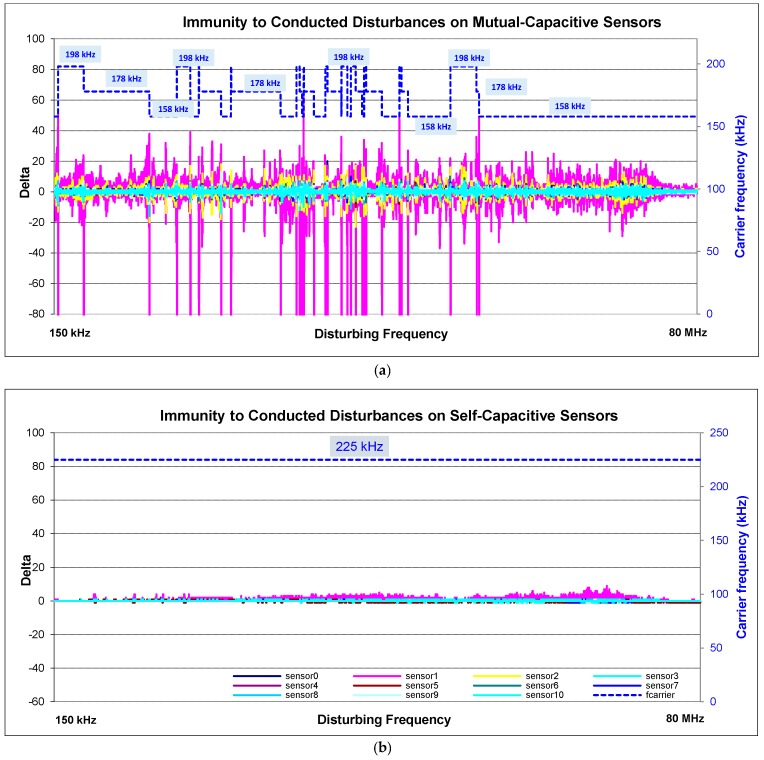
Conducted immunity test (**a**) frequency hopping is used and the carrier frequency varied between 158 kHz and 198 kHz when noise ratio reaches 3 and over; (**b**) on self-capacitance sensors.

The effect is shown in Figure 14a (also Figure 7 shows the results without frequency hopping). When the carrier frequency or its harmonics overlap with the noisy frequency, the sensors are disrupted. This effect is decreasingly ongoing on high carrier harmonics. Whenever the noise ratio reaches to 3, the carrier alters and the noise decreases. Self-capacitive sensors are less affected by the conducted immunity (Figure 14b). The pink sensor is the slider which consist of six sensing channels.

It was observed that self-capacitive sensors show greater immunity to EMC tests. They have their own sampling capacitors and are driven individually, not matrix. So, the measurement circuits do not interact. The simple electrode shapes may be another advantage. Despite showing better immunity, mutual-capacitance sensors can also be used safely thanks to frequency hopping and filters. The results obtained throughout the study are compared in Table 4 with hardware considerations.

## 6. Conclusions

The main contribution of this study was to present a new approach dealing with conducted noise in EMC tests. The advantage of the proposed method is that it does not require complex design. It is applied without using any external interface circuit, a component or an internal MCU module. This makes it cheaper and applicable to virtually all MCUs. We first detect the conducted noise by means of a noise detection algorithm which makes use of close correlation between disturbed signals for 16 sensor channels. Therefore, its memory consumption is low and the execution speed is quite high. It can detect the noise rapidly before causing false detection. Once the noise is detected, frequency hopping is successfully applied which changes the driving frequency so that the sensors are driven to a certain clearer band. The hopping is realized by an internal RC oscillator instead of using customized modules and it drives the sensors in narrow undisturbed bands. In current methods, instead of detecting the noise, sensors are driven in a widespread frequency band to reduce the noise effect overall so this generally needs an analog-digital circuit or customized modules.

Additionally, two touch sensing cards are designed using the charge-transfer method and by comparing them under several conditions to determine which one shows greater immunity to ambient conditions. The tests showed that the self-capacitive sensors show greater immunity to the EMC test. Also the signal levels are more stable in a noiseless environment. The mutual-capacitance sensors are more immune to temperature and water tests. It does not present a good enough EMC performance. When the noisy frequency overlaps with the driving frequency or its harmonics, interference increases and the sensors are affected. Therefore, the method was successfully applied and showed that this approach helps mutual-capacitance sensors to operate more reliably in a noisy environment and to succeed in EMC. The tests results are revealed graphically.

## Figures and Tables

**Table 1:** Sequence diagram of the mutual capacitance sensor circuit.

	*S*_1_	*S*_2_	*S*_3_	*S*_4_	NOTES
#1	Close	Open	Close	Close	*C_x_* and *C_s_* discharge
#2	Open	Open	Open	Close	Float State
#3	Open	Close	Open	Open	Pre-charge X-line
#4	Open	Close	Close	Open	Charge transfer
#5	Open	Close	Open	Open	Float State
#6	Close	Close	Open	Open	Isolate *C_s_* charge
#7	Close	Open	Open	Close	Discharge *C_x_*

**Table 2:** Sequence diagram of the self capacitance sensor circuit.

	*S*_1_	*S*_2_	*S*_3_	NOTES
#1	Close	Open	Close	*C_x_* and *C_s_* discharge
#2	Open	Open	Open	Float *C_s_*
#3	Open	Close	Open	Charge transfer
#4	Open	Open	Close	Float *C_s_* and settling time
#5	Close	Open	Open	Measure *V_Cs_* and discharge *C_x_*

**Table 3:** Carrier frequencies for mutual-capacitance sensors.

Carrier fc (kHz)	RCclk (MHz)	OSCCAL (kHz)
f_c1_: 158	8	155
f_c2_: 178	9	179
f_c3_: 198	10	198

**Table 4:** Comparison of mutual and self-capacitance sensors.

	Mutual Capacitance Sensors	Self-Capacitance Sensors
**Plus**	Needs fewer pins	Simple electrode design
	Better drop-let effect	Smaller code size
	Naturally moisture suppression	Fewer chip resource used
	Short burst—lower power consumption	Do not need to any reference point
	More localized touch-sensitive area	Stable, no crosstalk, more immune to EMC
**Minus**	More complex electrode design	Needs more pins Longer burst time More sensitive to temperature
	Larger code size	
	More chip resources used	
	Noise-sensitive	
	Unstable, crosstalk, EMC disturbance	
	Needs a reference point	

**Figure A1:**
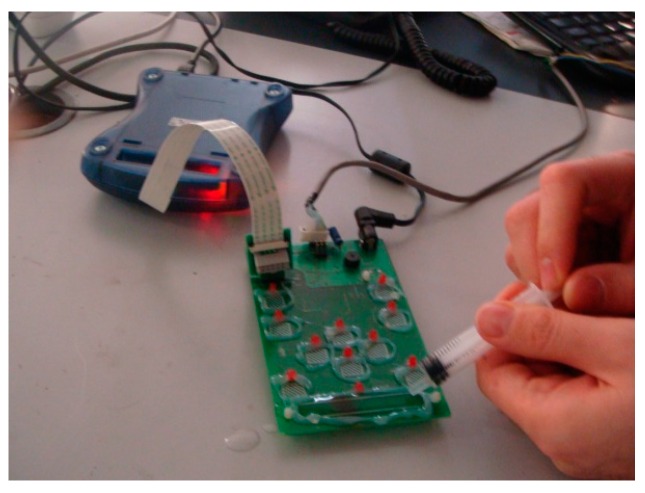
Mutual-capacitance.

**Figure A2:**
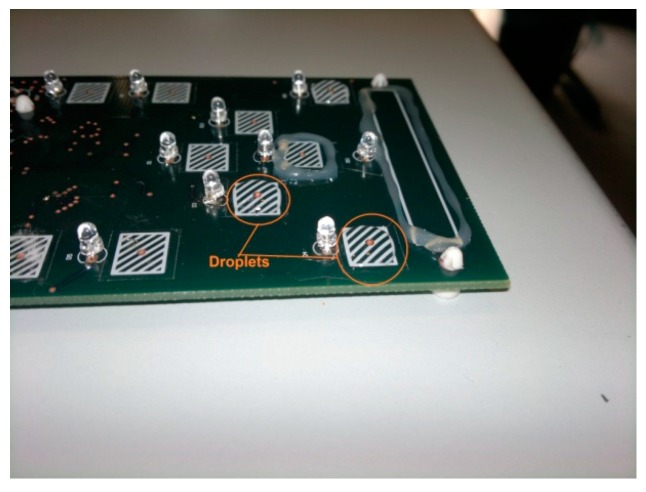
Self-capacitance.

**Figure B1:**
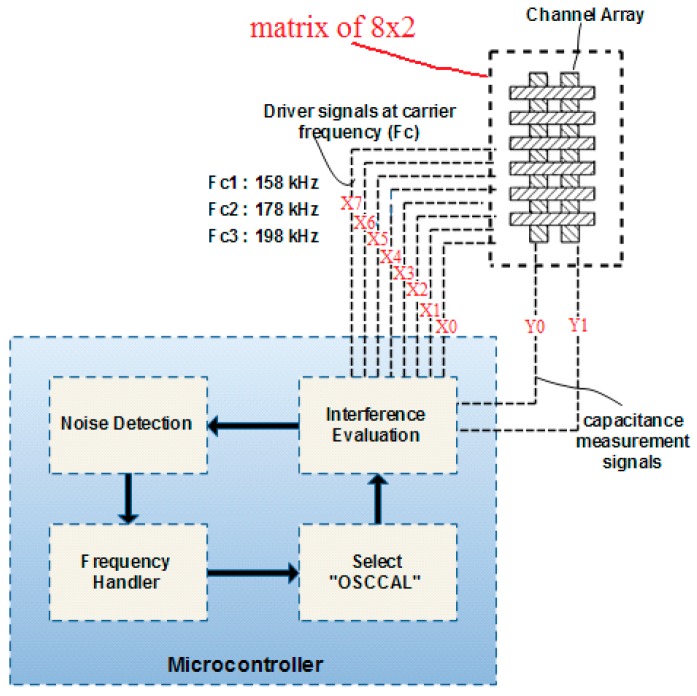
Frequency hopping approach based on capacitive sensors with interference rejection.

**Figure B2:**
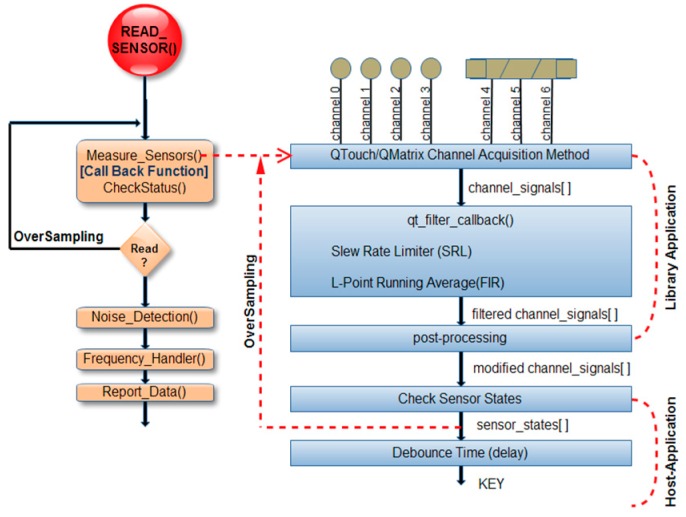
READ_SENSOR function with noise_detection and frequency_handler functions.

**Figure B3:**
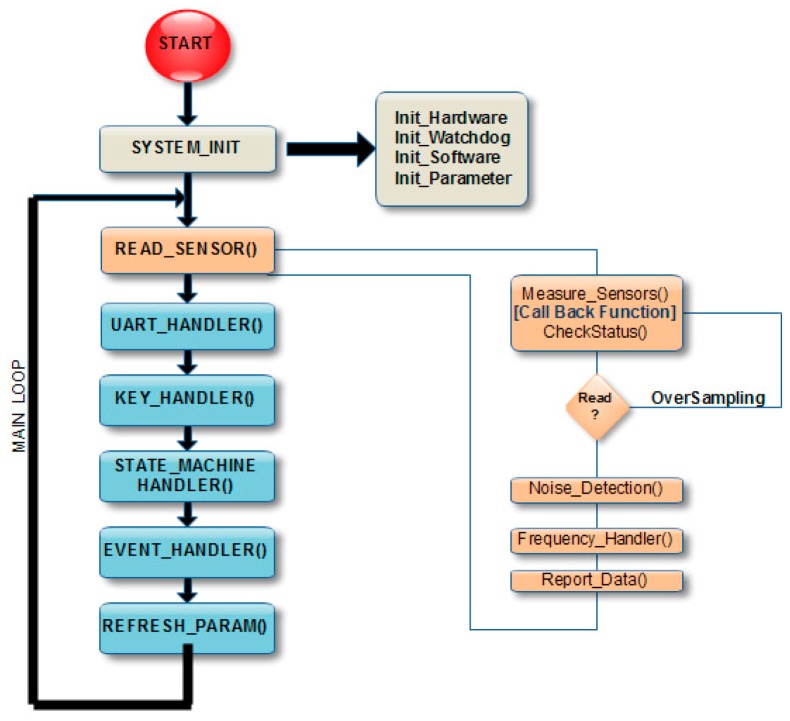
The flowchart of the main loop.

**Figure B4:**
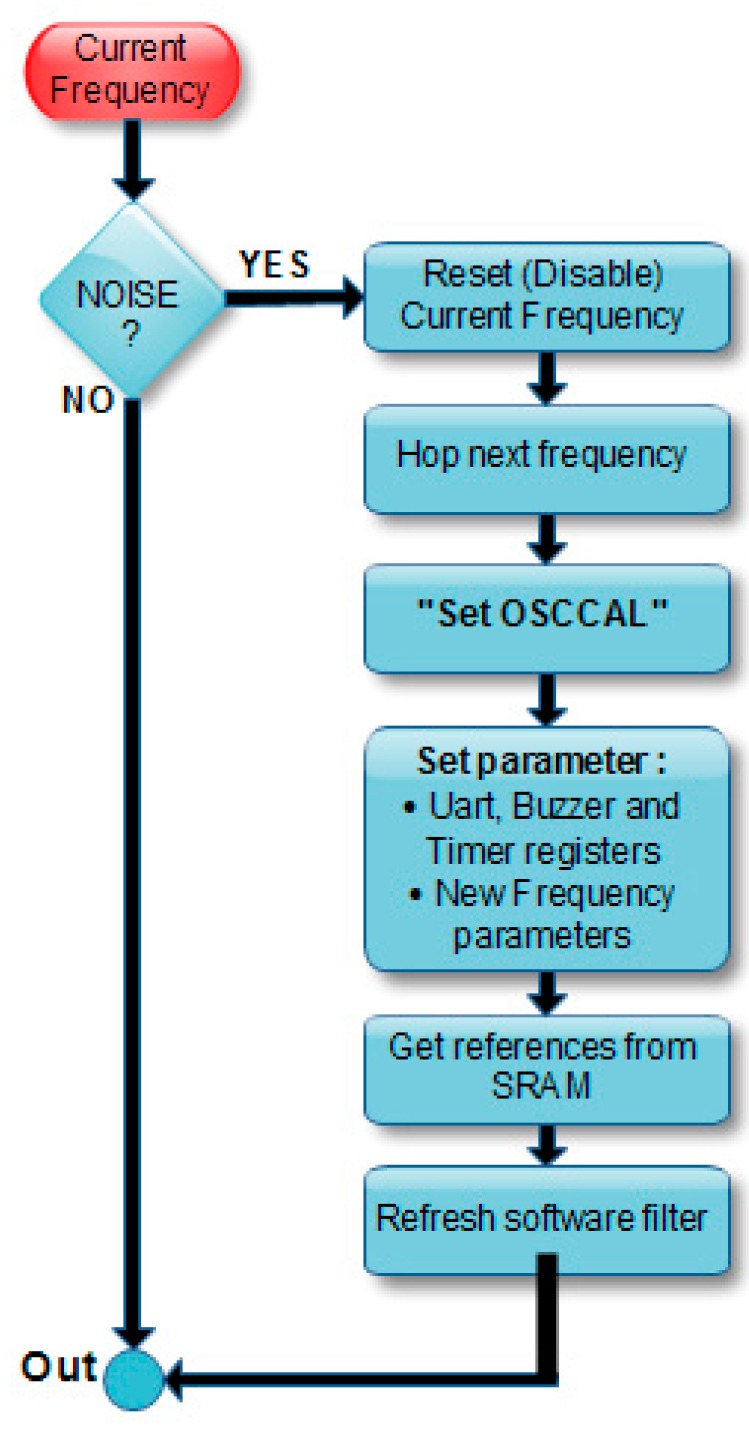
The flowchart of the frequency_handler.

## Appendix B

Both cards have exactly the same flowcharts except that frequency hopping functions in mutual-capacitive software.

## Appendix C. IEC 61000-4-6 Test

In this test, noise comes from power grids. The voltage slightly fluctuates and is quite difficult to be filtered. A sinusoidal signal (3 V) is modulated at 1 kHz. Later, a modulated signal is put on the power signal (220 V). Every 3 s, the modulation frequency increases 1% from 150 kHz–80 MHz. Some test parameters are:

- Frequency range: 150 kHz–80 MHz
- Voltage level (EMF): ±3 V
- Modulation: 80% (AM)
- Modulation frequency: 1 kHz
- Frequency step: 1%/3 s
